# The Dependence on Hue, Value and Opacity of Real-Time- and Post-Curing Light Transmission in a Nano-Hybrid Ormocer

**DOI:** 10.3390/ma17020496

**Published:** 2024-01-20

**Authors:** Nicoleta Ilie

**Affiliations:** Department of Conservative Dentistry and Periodontology, University Hospital, Ludwig-Maximilians-University, Goethestr. 70, D-80336 Munich, Germany; nilie@dent.med.uni-muenchen.de

**Keywords:** resin-based composites, optical parameters, shade, hue, value, exposure distance

## Abstract

This study aims to quantify the influence of hue, value and opacity on the variation in light transmittance of a full color palette of an ormocer-based dental composite. Samples with a thickness of 2 mm were cured in real time while the incident irradiance and light transmittance were recorded with a spectrophotometer, either in real time during the polymerization or through the polymerized composite at different exposure distances. Across the entire shade range, light attenuation was high, varying between 70.3% and 92.1%. The light transmittance during polymerization increased exponentially with exposure time in all shades. The differences between the cured and uncured composites decrease with increasing value and with increasing opacity within a value. The pattern of variation in light transmittance with increasing value is non-linear and depends on the hue, but not on the opacity within a hue. Small variations in value in lighter shades of hue B reduce the transmitted light more than in hue A, while the opposite is true for darker shades. The results strongly suggest an adequate curing of the lower increments in larger restorations, as the additional light expected from curing the upper increments is very small, regardless of hue, value or opacity. An additional unfavorable condition by increasing the exposure distance consistently contributes to a reduction in light transmission and thus further supports the above statement.

## 1. Introduction

In recent years, light-curing polymer-based composites have undergone various changes in composition, microstructure and implicit properties, with the trend clearly moving towards nanostructured hybrid materials. Such changes also raise the question of whether translucency and aesthetics have been appropriately adjusted to ensure unrestricted acceptance for practitioners and patients. These aspects are not only important for the aesthetic adaptation to the individual tooth situation and the patient, but play a crucial role in ensuring sufficient polymerization of the material in the depth of a restoration [1]. When insufficient translucency prevails, the lack of light reaching deeper layers of the restoration can have deleterious consequences for the patient and the longevity of the restoration, resulting in a low degree of monomer conversion [2], the elution of unreacted monomers [2], high toxicity [3] and hypersensitivity, as well as insufficient mechanical and chemical stability.

Ormocer-based composites are one such dental material with a fundamentally changed composition. Ormocers, the name being an acronym for organically modified ceramics [4], found their way into the dental industry after being successful in other industries [4] as, e.g., coating materials. They belong to the group of inorganic–organic hybrid polymers [4] and were developed more than 30 years ago at the Fraunhofer ISC (Fraunhofer Institut für Silicatforschung, Germany). In particular, their increased abrasion resistance [5,6], biocompatibility and ability to be synthesized with little or no shrinkage [7] make them an exciting source of inspiration for the development of novel dental materials.

Ormocers consist of pre-crosslinked inorganic networks based on polysiloxanes that are produced using the sol–gel process [8]. This inorganic Si-O-Si network, produced through hydrolysis and polycondensation reactions, is cross-linked by multifunctional urethane and thioether (meth)acrylate alkoxysilanes in the polymerization reaction [9].

Initial attempts to introduce ormocers into commercial composites encountered prototype upscaling and handling issues that forced the additional use of conventional methacrylates, thus reducing the above-mentioned advantages. Nonetheless, these first-generation ormocer-based composites were proven to generate a lower wear rate compared with conventional ones [5,6] and a shrinkage equal to that of hybrid composites, despite having less filler content [10].

As a material category, with the limitation of the low amount of commercially available ormocer-based composites, the mechanical properties do not differ compared to the hybrid composites [11]. The literature reports are somehow inconsistent for other parameters, which is due to the smaller number of analyzed materials, as in some studies, they have attested higher resistance to solvent degradation [12] and superior biocompatibility [13], but others remark on higher hydrolytic and fatigue-related degradation [14] when compared to hybrid or nano-hybrid composites. Likewise, they are recommended as restorative materials for posterior restorations due to their better fracture resistance and durability compared to nanohybrid composite restorations [15], but have shown lower clinical longevity in posterior restorations in a similar comparison [16]. It should also be noted that the clinical behavior of posterior restorations appears to vary between the use of ormocers in conventional, 2 mm layered composites, where they seemed to perform inferior to nanofill and nanohybrid RBC restorations, or in bulk-fill composites, where they perform similarly [16].

In addition to the summarized parameters and the clinical behavior described, the aesthetic aspect is no longer a negotiable compromise in modern dentistry, which is why the provision of a material in a wide range of colors is essential. Because this is achieved by adding, to a similar material formulation, dyes and pigments that absorb light [17,18,19], the light transmitted through a given material thickness can differ essentially among shades. If, in addition to absorption, light reflection and scattering are also taken into account, the light attenuation when passing through 2 mm thick samples made of CAD/CAM composites and ceramics is very high, with values between 59.9% and 94.9% related to the incident light [20]. Light attenuation through the tooth structure is also high under identical conditions and geometries and reaches a value of 89.4%, which is in the upper range of restorative materials [20]. All of these aspects raise concerns about whether the incident light from a light curing unit (LCU) can reach deeper layers of a composite, especially in darker or more opaque shades.

Therefore, the aim of the present study was to quantify, in real time, during the monomer-to-polymer transition, the variation in light attenuation of a full shade palette of an ormocer-based composite, while simulating its clinical use in 2 mm increments and using the light exposure conditions specified by the manufacturer.

The null hypotheses tested state that light transmittance and variation during exposure are similar across the shade palette, regardless of hue, value or opacity.

## 2. Materials and Methods

### 2.1. Materials

The entire color palette of an ormocer (organically modified ceramic) nano-hybrid resin-based composite (Admira Fusion, abbreviated AF, Vovo, Cuxhaven, Germany), which includes eighteen different variations of hue (A, B, C, D), value (1 to 4) and opacity (O), was analyzed with regard to real time light transmission during polymerization as well as light transmission through already-cured specimens. The filler weight fraction was 84% in all shades, and the matrix is described as ormocer-based. The following shades were analyzed: A1 (LOT: 1830320), A2 (LOT: 1831079), A3 (LOT: 1830088), A3.5 (LOT: 1829551), A4 (LOT: 1830417), B1 (LOT: 1828165), B2 (LOT: 1827574), B3 (LOT: 1824444), C2 (LOT: 1828384), D3 (LOT: 1826163), BL (LOT: 1828509), Inc (LOT: 1828196), GA3.25 (LOT: 1825051), OA1 (LOT: 1826435), OA2 (LOT: 1831331), OA3 (LOT: 1830478), OA3.5 (LOT: 1826556) and GA5 (LOT: 1828401). Light curing was carried out according to the manufacturer’s instructions, which was 20 s for shades A1 to GA3.25 and 40 s for the following materials in the sequence described above.

Incident and transmitted light was recorded with a spectrophotometer while using an LED (light-emitting diode) LCU (light curing unit) (Bluephase^®^ Style, Ivoclar Vivadent, Schaan, Lichtenstein). Incident and transmitted light were used to assess the kinetic of the light transmittance or to calculate further optical parameters.

### 2.2. Methods

#### 2.2.1. Spectrophotometry

Measurements were performed on a laboratory-grade USB4000 spectrometer (MARC (Managing Accurate Resin Curing) System, Blue light Analytics Inc., Halifax, NS, Canada), referenced by the National Institute of Standards and Technology (NIST), calibrated with an Ocean Optics’ NIST-traceable light source (300–1050 nm). The sensor used was 3.9 mm in diameter; consequently, only light that reached this area was taken into account.

The spectrometer uses a 3648-element linear charge-coupled array detector (CCD) with high-speed electronics (Ocean optic, Largo, FL, USA). Emitted light was collected over a 180° field of view by means of a CC3-UV Cosine Corrector (Ocean optic, Largo, FL, USA). Irradiances in a wavelength range of 360–540 nm were recorded individually at a rate of 16 recordings/s. The sensor was triggered at 20 mW.

*(a)*  *LCU characteristics*

The LCU’s irradiance and emission spectra was determined in five occasions by placing the LCU directly, centered and perpendicular to the spectrophotometer sensor. The measured values correspond to the incident irradiance, i.e., the irradiance that hits the sample surface. Incident irradiance was determined throughout the exposure protocol to assess the constancy of light emission over time.

*(b)*  *Light transmission*

Light transmittance was measured in real time during the light exposure of the material, which was carried out according to the manufacturer’s recommendations. Therefore, the materials were placed into cylindrical white plastic (polyoxymethylene) molds that were placed directly above the spectrophotometer sensor. The internal geometry of the molds, corresponding to the composite samples, had a diameter of 6 mm and a thickness of 2 mm. Five specimens were created for each shade. The LCU used for polymerization was positioned directly above, vertically and centered on the sample surface using a mechanical arm, while simultaneously aligned with the sensor below. The irradiance transmitted through each sample was measured in real time throughout the exposure period.

The measured incident and transmitted irradiance was then used to calculate the transmission in each shade. In addition, the exposure distance was varied in 2 mm steps up to 10 mm. Transmittance (T) was calculated as the ratio of transmitted (I_t_) irradiance (radiant power) to incident irradiance (I_0_), T = I_t_/I_0_, and is a dimensionless parameter.

#### 2.2.2. Statistical Analyses

The distribution of the variables was tested using the Shapiro–Wilk method. Since the variables were normally distributed, a parametric approach followed. One- and multiple-way analysis of variance (ANOVA) and Tukey honestly significant difference (HSD) post hoc-test using an alpha risk set at 5% was used (SPSS Inc. Version 29.0, Chicago, IL, USA).

## 3. Results

### 3.1. Emission Spectra of the Used LCU

**Figure 1:**
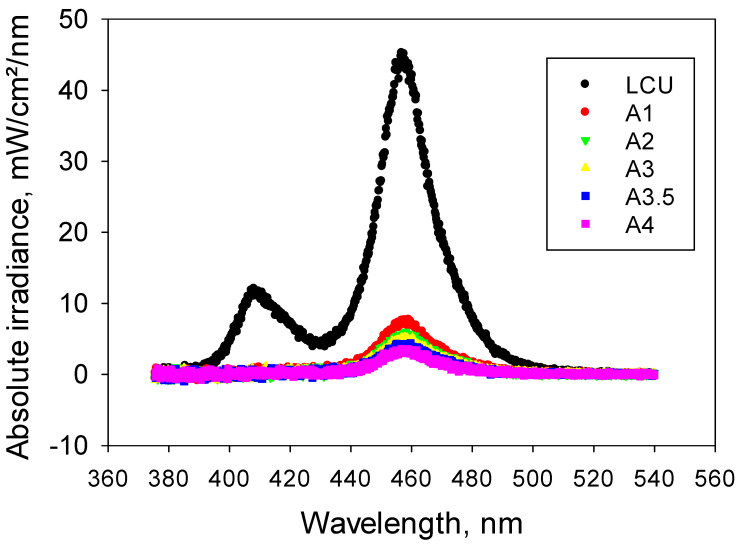
Emission spectrum of the LCU used and the spectra of the transmitted light measured at the bottom of 2 mm thick increments, exemplified for hue A (A1 to A4).

A violet (peak at 408 nm)–blue (peak at 458 nm) LED LCU was employed. The spectrum of the incident light is shown in Figure 1 along with the spectra of the transmitted light through 2 mm thick samples of shades A1 to A4. The irradiance of the incident light over the entire light spectrum was (1407.9 ± 12.1) mW/cm^2^. The incident light was strongly attenuated when passing through 2 mm thick RBC increments, as shown in Figure 1 for shades A, and the darker the shade (=the higher the value), the more attenuated the irradiance was. Details of the full color palette results are summarized in the images below.

### 3.2. Real Time Light Transmission during Polymerization in 2 mm Thick Increments

**Figure 2:**
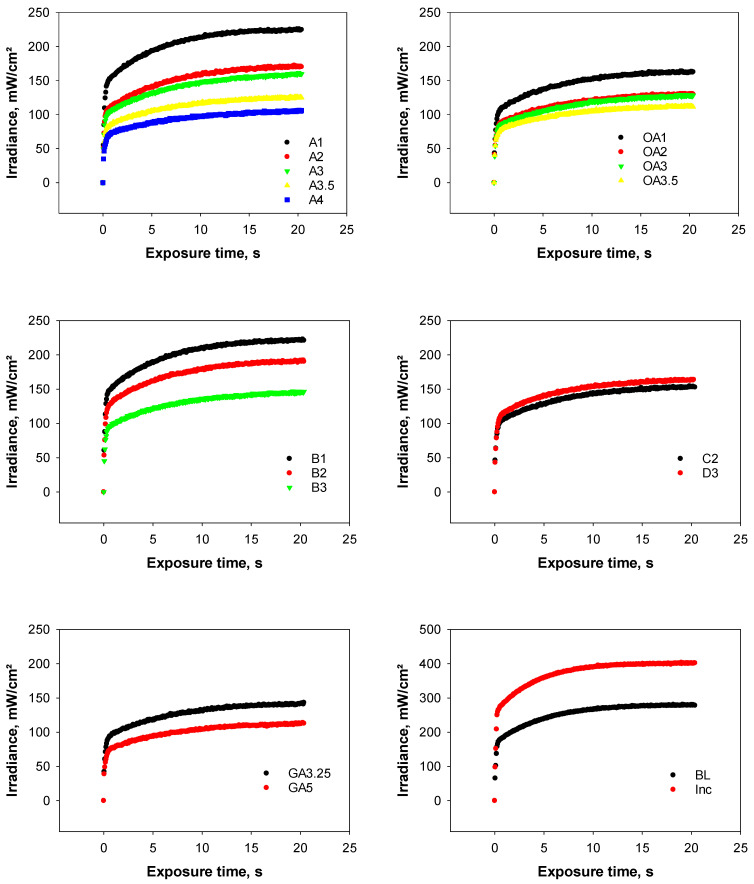
Transmitted irradiance (mean values) measured in real time through 2 mm thick composite samples of the 18 shades analyzed, while the LCU is positioned directly and centered over the top of the sample surface.

The real time light transmission whilst polymerizing the analyzed shade palette indicated an exponential increase in translucency with the increased polymerization time for all shades (Figure 2). The light attenuation through the 2 mm thick increments was high across all shades and varied at the end of the exposure from 70.3% (Inc) to 92.1% related to the incident irradiance of 1407.9 ± 12.1 mW/cm^2^ that reached the top of the specimens.

**Figure 3:**
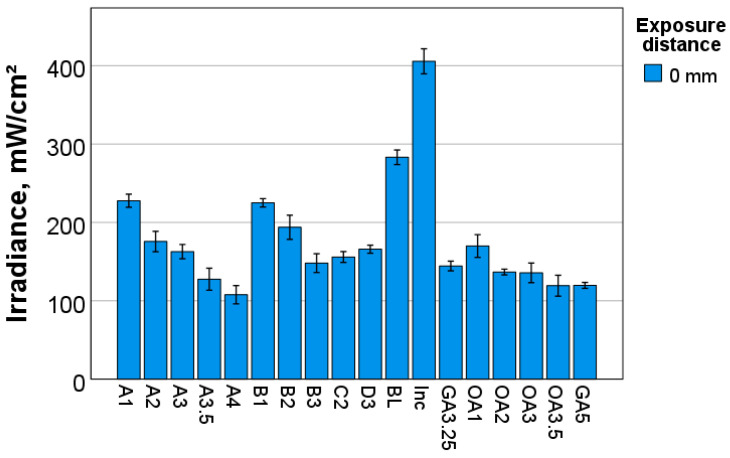
Transmitted irradiance at the end of the curing protocol (either 20 s or 40 s) as a function of shade when the LCU was positioned directly over the sample surface. The irradiance of the incident light was 1407.9 ± 12.1 mW/cm^2^. In each color tone (hue), a clear decrease in transmitted irradiance with value was observed.

The transmitted irradiance measured at the end of the curing protocol is summarized in Figure 3. The significantly highest light transmittance was recorded for Inc and decrease in the sequence: Inc > BL > group of A1, B1 (*p* = 0.999) > B2 > group of A2, A3, D3, OA1 (*p* = 0.064) > group of A3, C2, D3 (*p* = 0.348) > group of B3, C2, GA3.25 (*p* = 0.158) > group of OA2, OA3, B3, GA3.25 (*p* = 0.09) > group of A3.5, OA2, OA3 (*p* = 0.494) > group of A3.5, OA3.5, GA5 (*p* = 0.653) > group of A4, OA3.5, GA5 (*p* = 0.122).

**Figure 4:**
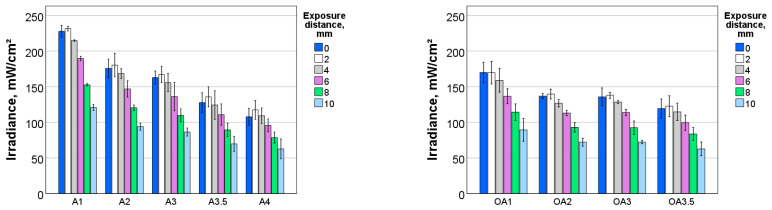

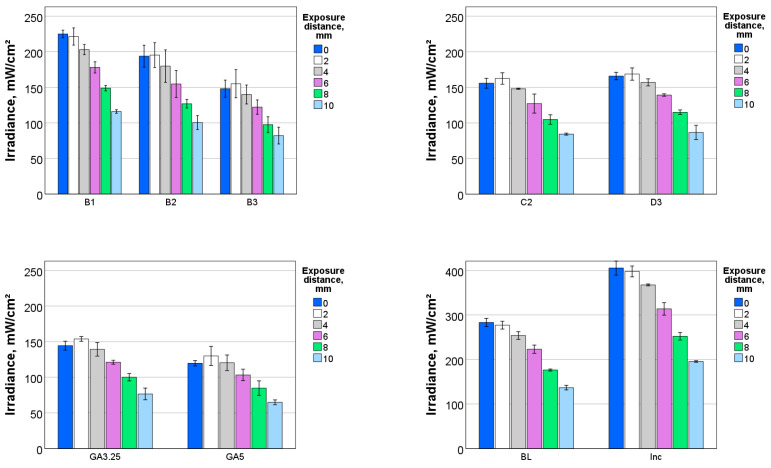
Transmitted irradiance (Ir) through cured 2 mm thick composite increments as a function of shade and exposure distance.

The light transmission through an already-cured 2 mm thick resin composite sample when the exposure distance was increased from 0 to 10 mm is summarized in Figure 4. At a short exposure distance of 2 mm, the transmitted irradiance values were maintained compared to placing the LCU directly above the sample surface, but decreased significantly as the exposure distance increased. The variation in transmitted irradiance was strongly influenced (*p* < 0.001) by both parameters—shade (partial eta-squared η_P_^2^ = 0.996) and exposure distance (η_P_^2^ = 0.989). The binary effect of the main parameters was also very strong (η_P_^2^ = 0.943).

**Figure 5:**
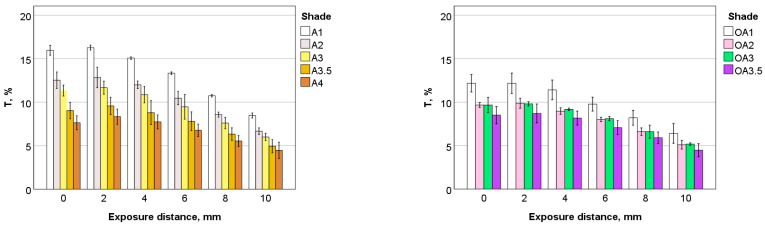

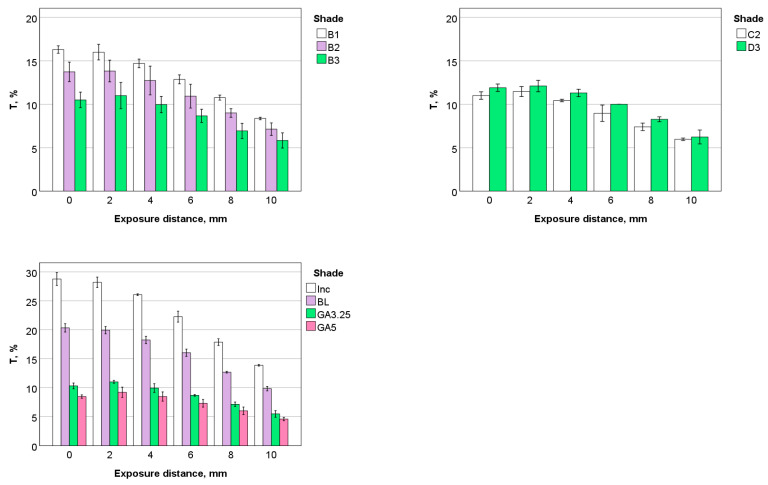
Percentage transmittance (T) through 2 mm thick composite increments as a function of shade and exposure distance.

The variation in transmittance as a function of hue, value, opacity and exposure distance is summarized in Figure 5. For the shade palette and curing conditions analysed, transmittance varied between 4.3% (A4, exposure distance 10 mm) and 28.7% (Inc, 0 mm exposure distance).

The pattern of transmission variation with brightness (value) variations appeared to be hue-dependent. In particular, for hue A and for both regular and opaque formulations, a significant decrease in transmittance was observed in lighter shades (A1 vs. A2), while the difference between the next brightness levels (A2 and A3) was small. A decrease in transmittance in darker shades from A3 to A3.5 and A4 followed. These differences were pronounced at smaller exposure distances and balanced out at larger exposure distances. The non-linear decrease in transmittance with brightness shown for hue A was not observed in hue B, where the decrease from brightness level one to two and two to three was comparable. By far the highest transmittance was observed for Inc, followed by BL with almost a third lower transmittance. Furthermore, when comparing hues A, B and C in the only common brightness level, two, the highest transmittance was observed in B, followed by A and then C.

## 4. Discussion

Coloring allows dentists to customize a composite material to match a patient’s tooth as closely as possible, providing a natural appearance for dental restorations. Although little is described in the literature about the technical implementation of coloring, it is by no means a trivial process in the production of a dental material. In addition to the classic method with pigments, coloring is also carried out through the targeted selection of complex structures that are fine enough to interfere with visible light (structural coloring), based on the example of nature [21]. Also, a combination of both methods is possible [21]. It is important to note that matching the color of restorative materials to natural teeth is not nearly as demanding as matching translucency, which is considered the main cause of aesthetic failure [22,23]. The selected ormocer-based composite therefore offers a large pallet of different shades, while varying the hue (A, B, C, D), value (one up to four) and translucency (translucent to opaque). All 18 shades of the color palette were comparatively evaluated in the present study with regard to their ability to transmit as much of the incident light as possible into the deeper layers of the material. Since light attenuation increases exponentially with material thickness according to Lambert–Beer’s law [24], a constant sample thickness of 2 mm was used to meet the clinical application requirements of the analyzed material.

Light transmittance monitored in real time during polymerization increased exponentially with exposure time in all shades, following the pattern observed to date in most commercially available composites [25,26]. The exposure time specified by the manufacturer and an LCU with the appropriate irradiance were used in all measurements. The differences between the cured and uncured materials decrease as the value increases and as the opacity increases within a value. The increase in translucency during the monomer-to-polymer transition is closely related to the increasingly improved match between the refractive index of the filler and the forming polymer [1,27,28]. Since the components responsible for absorption and reflection, such as the monomer matrix [18] and fillers [29,30], are similar in chemical composition and quantity in all shades, the measured differences in light transmittance between the 18 shades must be related directly to differences in color and opacity [17,18,19]. Therefore, a deeper dive into some more detailed considerations for coloring dental materials is required and is provided below.

While structural color results from selective light reflection, pigment color is created through selective light absorption by the electrons of pigments [31]. Structural color is a rather new type of coloring of dental materials and can be achieved by scaling the size and arrangement of the (nano)fillers embedded in the organic matrix [32]. Although it offers the advantages of being environmentally friendly and nonfading, it is a much more sophisticated approach [31] since it requires the creation of nanostructures capable of reflecting or scattering light, so that the waves of specific frequencies interfere constructively [33]. In addition, the nanostructures must be disordered to avoid the angular dependence of color perception [32]. Structural coloring has already been implemented in commercially available materials, primarily through the use of core–shell structures of uniform size (260 nm), where the core is made of either silica or zirconia and the shell is a polymer with a significantly lower refractive index compared to the core [34]. Color can be then adjusted by varying the thickness of the shell and thus implicitly the distance between the inorganic nanoparticles, while the opacity is adjusted based on the scattering strength of the particles [35]. The created nanostructure is described as producing a red-to-yellow color when ambient light passes through the material and allows for better color matching compared to pigmented colored RBCs [36,37].

Coloring with pigments is by far the most common method of imparting color and opacity to composites and was apparently used in the material analyzed, as a specific nanostructured arrangement of the filler as described above is not apparent. In addition to structural coloring and pigments, variations in the size and amount of inorganic fillers are also used to adjust opalescence and translucency in regular composites [38]. Pigments change the spectrum of the light they reflect by primarily absorbing parts of it. The color impression is thus created subtractively: a yellow pigment strongly reflects the color yellow while it swallows blue, whilst a blue one behaves the other way around. In addition to absorption, pigments are able to scatter light, which can affect color perception because there is a wavelength-dependent change in the direction of propagation. In this context, the size of the pigment particles plays an important role in the formation of color. If the particles are significantly smaller than the distance between two wave crests of the incident light, the scattering intensities are inversely proportional to the fourth power of the wavelength, as described by Rayleigh [39]. It was shown that the scattering in dental composites is highest when the diameter of the particles corresponds to approximately half the wavelength of the incident light, i.e., ~0.2–0.3 µm [1]. In contrast, particles with dimensions in the wavelength range and above scattered light are less dependent on the wavelength and also preferentially in the forward direction. It should also be taken into account that, due to their small dimensions, pigment particles have a large surface area in relation to their volume. This enables them to interact intensively with the embedding medium, sometimes preferably with individual components of it, and also with other particles. Therefore, agglomerations can occur and affect the properties of the color. The tendency to form these agglomerates, as well as their number and stability, depend on the chemical composition and structure of the pigment surfaces, their electrostatic charge, the prevailing temperature and the composition of the medium. For this reason, it is important to be able to influence the pigment–pigment interaction through a targeted modification of the surfaces.

Coloring with pigments is therefore an extremely complex process, since in order to optimize a pigment mixture in terms of scattering and absorption to induce a specific color property in a material, its interaction with visible light must first be empirically determined. It is therefore somehow understandable that there is no declared information about the amount and type of pigments used in dental composites to achieve the desired tooth color match. On a general note, white pigments or opacifiers are characterized by high scattering and low absorption in the entire visible wavelength range. Very suitable representatives are titanium dioxide (TiO_2_) [40], especially in the crystallographic modifications rutile and anatase (refractive index 2.8 and 2.55, respectively). They have optimal scattering performance, with a particle size of 0.19 µm and 0.24 µm. Effective opacifiers, in addition to TiO_2_, are zirconium oxide (ZrO_2_) and aluminum oxide (Al_2_O_3_) [17]. The colored pigments scatter and absorb depending on the wavelength, resulting in a wide variety of color gradations that can be characterized based on their reflection spectrum. Yellow-to-brown pigmentation is very often achieved using iron oxide pigments to produce hues of classification A, B, C or D, where A is reddish-brownish, B is reddish-yellowish, C is grayish and D is reddish-gray. Within each hue, the additional number indicated represents the value defined from the lightest value (one) to the darkest and most intense value (four). In addition, the palette contains four opaque dentine shades (OA1 to OA3.5), one translucent enamel shade (Inc), one very light, so-called bleach shade (BL) and two gingiva shades (GA). Although the type and amount of pigments or opacifiers are not known, the variation pattern of transmitted irradiance within the analyzed color palette confirms what was previously observed with another material [41], namely the lack of linearity of light transmission variation within a hue and clear differences in the variation pattern of a hue. A significantly greater variation in light transmittance was observed for shade A compared to shade B, with the result that shade B1 was more translucent than A1, but B3 was less translucent than the corresponding value, A3. In addition, the light transmittance in hue A decreases in smaller increments for lighter shades (step A1–A2) than for darker shades (step A2–A3), while the opposite is the case for hue B. This comparison cannot be made for hue C and D, as only one value is available for each of them. A systematic analysis of the variation in light transmittance for each hue requires experimental materials and a much larger variation in values and opacities within one hue. This would be the only way to accurately calculate the relationships between the amount and type of pigment and light transmission. The behavior observed in hues A and B can be well related to the type of pigmentation used, as hue B is reddish/yellow colored, with the yellow pigments better absorbing the blue light of the LCU during the curing of the material compared to the reddish-brownish pigmentation used in hue A. Across the entire color range, the differences in light attenuation for the ormocer-based composite vary between 70.3% for Inc and 92.1% for A4, thus being in the same range as the values found in a similar color range of a methacrylate-based, giomer composite (72% to 93.6% [41]).

The effect of opacity can be clearly observed with hue A, which offers an opaque equivalent to all translucent shades. The variation pattern of light transmittance with increasing value is preserved in the opaque series compared to what was described above for shade A, but the variation steps are much smaller. The opaque shades are less translucent than their more translucent version at the same value, but only up to a value of 3.5, where the difference is no longer significant. The darker values are therefore so strong in weakening light transmission in that additional opacification is either no longer applied or it no longer affects the light transmission.

Another clear finding of this study with high clinical relevance is that the light transmittance during the exposure of a 2 mm thick increment is very low, regardless of the hue, value or opacity used. This thickness was chosen to assess light transmission to replicate the clinical use of the material. In clinical dentistry, when incrementally restoring a larger cavity, it is sometimes assumed that it is sufficient to pre-cure the first, lowest increment for a few seconds, as the material receives additional light when curing the next, upper increments. This assumption must be expressly rejected because the light transmission in 2 mm increments is very low, even with the most translucent color (Inc). An additional unfavorable condition when increasing the exposure distance consistently contributes to a reduction in light transmission, and thus further supports the statement.

When designing this study, an attempt was made to replicate the clinical conditions as best as possible. The test specimens were polymerized in 2 mm increments according to the manufacturer’s instructions, and the recommended polymerization time, depending on the color of the material, was strictly adhered to. The mold used to test light transmission was a white plastic cylinder to mimic tooth color, as the internal dimension of the mold and the material have been shown to affect the depth of polymerization [42]. Unpublished internal data show that there is a slightly (approx. 5%) higher light transmission (through air) when using this mold compared to a mold consisting of a real tooth surrounded by a pink polymer that imitates the gingiva. Another influence on light transmission would certainly be the temperature at which the tests were carried out, as these were performed at room temperature and not at 37 °C. However, it should be noted that even in clinical use, the restorative materials cannot rise to body temperature in the short time between application in a cavity and light exposure, while the largest increase in temperature is caused by the LCU and the exothermic polymerization reaction. Since the clinical indication for composite placement requires a dry environment, the difference from laboratory testing should also be of little importance with regard to moisture.

## 5. Conclusions

Real time light transmission during polymerization emphasizes strong light attenuation (70.3% to 92.1%) while passing through 2 mm thick layers of material and has a strong dependence on hue, value and opacity, thus allowing the rejection of all null hypotheses. The pattern of variation in light transmittance with increasing value is nonlinear, dependent on hue but not on opacity within a hue. When varying the value from one to two in hue B, greater decreases in light transmission must be taken into account, while the differences in hue A for the same values are small. When varying the value from two to three, i.e., with increased darkness, the situation is reversed. Adequate curing of the lower increments is essential in the incremental restoration of a cavity.

## Data Availability

Data are contained within the article.
